# Municipality politicians’ self-rated knowledge of work environments for key professions within Swedish elder care

**DOI:** 10.1108/JHOM-12-2024-0489

**Published:** 2025-06-17

**Authors:** Susann Viktoria Porter, Ulrika Flädjemark, Per Schubert

**Affiliations:** Department of Health and Society, Faculty of Health and Society, Malmö University, Malmö, Sweden; Department of Urban Studies, Faculty of Culture and Society, Malmö University, Malmö, Sweden; Department of Natural Science, Mathematics and Society, Faculty of Education and Society, Malmö University, Malmö, Sweden

**Keywords:** Elder care, Elder care professionals, Municipality government, Municipality politicians, Work environment

## Abstract

**Purpose:**

Municipality politicians in Sweden represent their municipalities as employers, bearing overall legal responsibility for the work environment. This study examines the self-rated knowledge of politicians responsible for elder care in the settings of home care and special housing in Sweden. The study focuses on two aspects of their role: their knowledge regarding the work of the key professionals who deliver elder care services (including care assistants, assistant nurses, registered nurses, physiotherapists, occupational therapists and first-line managers) and their knowledge of their accountability as employers for the work environments.

**Design/methodology/approach:**

This quantitative study is based on self-reported responses to a questionnaire from 81 municipality politicians. All of Sweden’s 290 municipality political board chairs were invited to participate and asked to extend the invitation to other board members. The study is part of a larger study with a longitudinal single-group pre-post experimental research design that evaluated a digital educational programme regarding organisational and social work environments within elder care, targeting accountable municipality politicians in Sweden.

**Findings:**

Politicians may lack knowledge regarding the work of elder care professions and their legal responsibilities as employers. The level of knowledge was higher among board chairs compared to board members. Regardless of political role, knowledge of the work of care assistants and assistant nurses was highest, whilst that of physiotherapists and occupational therapists was lowest.

**Research limitations/implications:**

The results of this study are based on a questionnaire designed to assess the self-rated knowledge of municipality politicians regarding the work of key professions in elder care within special housing and home care contexts.

**Practical implications:**

These results highlight the knowledge imbalance between board chairs and board members and are significant because all board members share equal responsibility for the decisions made. Given the limited time board members have to fulfil their political roles, it is essential to consider this constraint when addressing their knowledge needs.

**Social implications:**

This study provides insights into the self-rated knowledge of municipality politicians responsible for elder care and focuses on two key aspects of their role. Examination of their knowledge of the work performed by key professionals who deliver elder care services indicate differences in knowledge levels based on political roles. Board chairs demonstrate a higher overall degree of knowledge compared to board members.

**Originality/value:**

This research examines the knowledge of municipality politicians accountable for elder care regarding the work of different key professions within elder care, in both special housing and home care contexts as well as their knowledge of work environment legal responsibilities.

## Introduction

In Sweden, democratically elected politicians govern healthcare provision at three levels: the government (i.e. national level) sets the overall healthcare agenda (SFS, 2017:30 Health and Medical Services Act), 21 regions are responsible for delivering healthcare at county level, and finally, 290 municipalities are responsible for providing elder care services as well as other services at municipality (i.e. local) level (SFS, 2017:725 Local Government Act). In Sweden, the regions and municipalities have governing decision-making power over healthcare within their respective areas (SFS, 2017:30 Health and Medical Services Act). This paper engages specifically with healthcare organised by the municipalities, namely elder care in various settings.

Two main laws regulate elder care service in Sweden: The Health and Medical Services Act of 2017 (SFS, 2017:30 Health and Medical Services Act) and the Social Services Act of 2001 (SFS, 2001:453 Social Services Act). The Health and Medical Services Act states that medical care should be provided to those in need. Whilst the Social Services Act specifies that the elderly should be able to live dignified lives and have well-being and that municipalities should work to ensure that elderly citizens can live independently, in safe conditions, with an active and meaningful existence in community with others. The elderly are entitled to support and care either at home or in special housing, according to their needs (SFS, 2001:453 Social Services Act). The municipalities of Sweden are both the main providers and direct employers for elder care services (Swedish Association of Local Authorities and Regions, 2021).

There are approximately 34,500 politicians with various political roles in the municipalities within Sweden (Statistics Sweden, 2024). The municipality councils independently select the boards and committees that govern the various areas of responsibility, and therefore, board and committee structures can differ between municipalities. As politicians represent the municipality as employers, they have the ultimate responsibility for the work environments of the various professions within their area of accountability (Swedish Association of Local Authorities and Regions, 2019a, b). A political board chair has specific obligations to convene board meetings, conduct the meetings and prepare minutes. The board chairs and the board members each have a single vote and are equally responsible for decisions made (SFS, 2017:725 Local Government Act, Swedish Association of Local Authorities and Regions, 2019a).

The Swedish Work Environment Act (SFS, 1977:1160) specifies that employers have legal responsibility for the physical and psychosocial work environment of their employees, including the requirement to eliminate or mitigate risks in the work environment that could lead to ill health (Swedish Work Environment Authority, 2019; Ahlberg, 2018). The present study examines the self-rated knowledge regarding the municipality politicians’ accountability as employers for the work environment. The study focuses furthermore on their knowledge of the work performed by the key professionals who deliver elder care services, including care assistants, assistant nurses, registered nurses, physiotherapists, occupational therapists and first-line managers. According to the Work Environment Act, employers can delegate work environment responsibilities to other actors in the organisation, i.e. civil servants and first-line managers (SFS, 2017:725 Local Government Act). However, the ultimate work environment responsibility cannot be delegated and employers must make sure that those who receive the delegation have the knowledge and resources to execute the task (SFS, 1977:1160 Swedish Work Environment Act).

Taking this into account, previous research on the municipality politicians’ responsibility for Swedish elder care shows that politicians may lack knowledge of their responsibility as employers for the work environment, including knowledge of the work done by different elder care professions (Porter and Muhonen, 2021; Porter, 2022).

The most common professionals working in elder care in Sweden are assistant nurses, which is also the most common profession among women in Sweden. In 2023, approximately 128,000 assistant nurses (112,110 women) and 95,070 care assistants (67,950 women) were employed in elder care (Statistics Sweden, 2025). The municipality provides nursing and rehabilitation services through registered nurses, occupational therapists and physiotherapists (Swedish Association of Local Authorities and Regions, 2023; National Board of Health and Welfare, 2025). First-line managers in elder care are each responsible for an average of 48 employees, which is twice as many employees as the average manager in the Swedish labour market (Institute of Stress Medicine, 2022).

The municipality should provide good quality care for the elderly, although work-related stress among the staff is common and can affect care quality and provided support (Foà *et al.*, 2020; Daly and Szebehely, 2012; Josefsson, 2012). Stress of conscience occurs when staff cannot provide the desired care for their elder care recipients (Alkrisat and Alatrash, 2016; Åhlin *et al.*, 2022; Glasberg *et al.*, 2006). Research has shown that care givers within home care are exposed to higher levels of stress of conscience than those working within the special housing setting (Åhlin *et al.*, 2022). Additionally, lack of adequate time to deliver the desired care to the elderly can negatively affect employee health (Daly and Szebehely, 2012; Josefsson, 2012). Sick leave due to mental health problems is prevalent among staff working in elder care (Swedish Social Insurance Agency, 2020a; Swedish Association of Local Authorities and Regions, 2019a, b; 2012), and a literature review shows that staff burnout is a significant problem (Harrad and Sulla, 2018).

First-line managers in elder care are crucial for influencing the staff’s work environments. An empowering leadership that supports staff is associated with a more positive psychosocial work environment (Lundgren *et al.*, 2016). Adding to the evidence on the importance of a positive work environment, research has shown that a psychosocial work environment perceived as healthier by staff can lead to higher satisfaction among the elderly receiving care (Lundgren *et al.*, 2016). However, first-line managers in elder care can perceive their work to be both complex and lacking the necessary organisational support. Their responsibilities reflect strained financial resources for elder care and responsibility for many employees (Hagerman *et al.*, 2019).

In the Swedish governmental model, politicians and civil servants work collaboratively. Decision material for boards and committees is provided by the civil service administration and must be factual, objective and politically impartial (Christensen and Opstrup, 2018; Swedish Association of Local Authorities and Regions, 2019a). The civil servants’ primary responsibility is to implement political decisions (Christensen and Opstrup, 2018; Swedish Association of Local Authorities and Regions, 2019a). The heads of administration within the civil service are responsible for ensuring that organisation of work is conducted in accordance with formulated goals and guidelines and that budget frameworks are kept (Swedish Association of Local Authorities and Regions, 2019a). Importantly, the outermost accountability lies within the politicians’ area of responsibility, and it is from here the overall organisational goals emerge.

### Systematic Work Environment Management – SWEM

**Figure 1:**
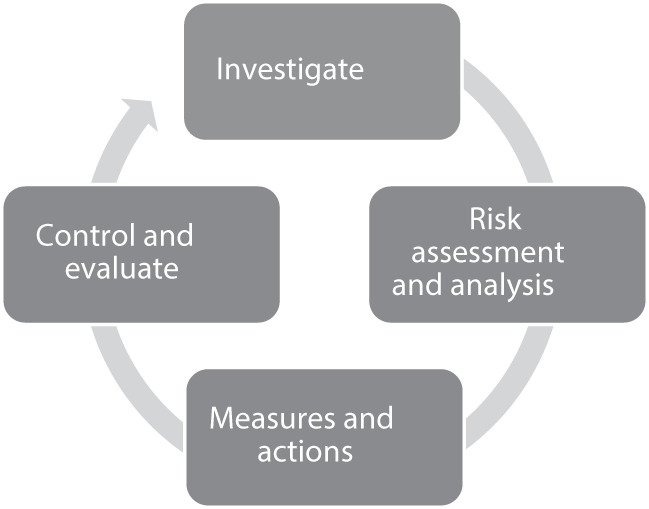
The Systematic Work Environment Management – SWEM. Source: Authors’ own work derived from information on the Swedish Work Environment Authority’s official website and AFS (2001:1) Systematic Work Environment Management [Provision (2001:1) Systematic Work Environment Management]

An important aspect of complying with the Swedish work environment legislation is the Systematic Work Environment Management (SWEM) methodology, which also is a provision, i.e. binding rules within the work environmental legislative framework (AFS, 2001:1 Systematic Work Environment Management). This provision emphasizes that all employers have a responsibility to investigate the work environment, perform a risk assessment and take measures to eliminate or reduce risks. Employers are also responsible, at least annually, to control that the measures have had the desired effect and that new risks have not arisen (Figure 1). Therefore, the SWEM methodology is a continuously ongoing procedure, often called “the SWEM-wheel”. SWEM covers all employers in Sweden and all parts of the work environment, and a prerequisite for applying SWEM is a good understanding from the employer of the employees’ work and work environments (AFS, 2001:1 Systematic Work Environment Management).

Accountable politicians must ensure that their responsible areas include work environment in accordance with the Working Environment Act (SFS, 1977:1160). They should allocate SWEM actions to the administrative managers and ensure that the managers have sufficient resources and knowledge to conduct this work. They should also ensure that preventive measures in the work environment can be taken by including work environment investments in the annual budget proposal and ensure a review at least once during the year. If responsible politicians make significant decisions, for example, to reduce the number of staff, a written risk assessment must first be made (Swedish Association of Local Authorities and Regions, 2022).

Previous research shows a willingness among politicians to enhance their own knowledge of their area of responsibility, although this goal can be difficult to achieve because of the limited time available (Porter, 2022). Most politicians have other jobs and areas of responsibilities that require their attention (Statistics Sweden, 2024). The knowledge deficit, along with limited time spent on their board assignments, could result in a knowledge imbalance between civil servants and politicians that impacts collaboration within the governance framework (Porter, 2022). The current study examines the self-rated knowledge of municipality politicians responsible for elder care in Sweden. This is accordingly an empirical analysis based on real-life direct measurement investigating specific settings. Two aspects of a municipality politician’s role are evaluated. First, their knowledge of the work of the key professions that deliver elder care services, and second, their knowledge regarding their accountability as employers for the work environments.

## Method

### Research design

This study is part of a larger empirical study with a longitudinal single-group pre-post experimental research design (Shadish *et al.*, 2002) that employed a pretest–posttest approach to evaluate a digital educational programme regarding organisational and social work environments within elder care, targeting accountable municipality politicians in Sweden. This study explores participant knowledge prior to undertaking the digital educational programme. Data collection began in January 2024 and ended in October 2024, including the data collection for the larger study.

### Participants

Participants were eligible for the study if they were a politician elected to a municipality board responsible for elder care, were at least 18 years old, and could communicate in Swedish. The participants were recruited by the study project leader who contacted all board chairs with responsibility for elder care across Sweden’s 290 municipalities. Board chairs were sent study information and a consent form by email. The board chairs were encouraged to distribute the invitation to the other board members. Due to initial recruitment difficulties, invitations were also extended directly to board vice-chairs, deputy board vice-chairs and board members. The study aimed to have a comprehensive design with the assistance of the board chairs. However, because there was no guarantee that all board members received the invitation to participate, this was not feasible. In total, 1,556 emails, including reminders, were sent to potential participants. Four participants actively declined to participate and provided explanations of high workload (*n* = 1), no remuneration for study participation (*n* = 2) and already having enough knowledge (*n* = 1). The recruitment concluded with 81 participants, including board chairs, board vice-chairs, deputy board vice-chairs, board members, deputy board members and one participant who did not specify their board role (Table 1).

**Table 1:** Characteristics of the participating municipality politicians (*N* = 81)

Characteristics	*n*	%	Years (mean/range)
Age	80	98.77	60.3/33–91
*Missing value*	1	1.23	
Female/Male	51/30	62.96/37.04	
Geographic region of Sweden			
Götaland	37	45.68	
Svealand	35	43.21	
Norrland	8	9.88	
*Missing value*	1	1.23	
Educational level			
Upper secondary	22	27.16	
University	59	72.84	
Political party			
The Moderates	26	32.10	
The Social Democrats	20	24.69	
The Christian Democrats	9	11.11	
The Left Party	11	13.58	
The Centre Party	4	4.94	
The Liberals	3	3.70	
The Swedish Democrats	3	3.70	
The Green Party	2	2.47	
Local independent parties	3	3.70	
Political assignment for the elder care			
Board chair	23	28.40	
Board vice-chair	12	14.81	
Deputy board vice-chair	10	12.35	
Board member	23	28.40	
Deputy board member	12	14.81	
*Missing value*	1	1.23	
Years as responsible politician for			
Elder care	76	93.83	4.7/1–30
*Missing value*	5	6.17	
Work environment training received during the last two years			
Yes	18	22.22	
No	63	77.78	

**Source(s):** Authors’ own work

### Ethical considerations

This study was conducted in accordance with the ethical guidelines of the *Declaration of Helsinki – Ethical Principles for Medical Research Involving Human Subjects* (World Medical Association, 2001). Written information concerning the study aim and a consent form were emailed to potential participants. Written consent was obtained from each study participant by email or post to the project leader. All emailed consents were printed out and stored in a locked safe box, and the original emails were deleted. Confidentiality and the right for participants to terminate without giving a cause were guaranteed. This study was approved by the Regional Ethical Board in Linköping, Sweden (Dnr, 2022-01999-01).

### Data collection

Baseline characteristics were collected via a web-based questionnaire developed in the web application REDCap, which is used for creating and distributing surveys and questionnaires for research projects via the Internet (REDCap, 2024). Participants received the questionnaire through an email link. Collected demographic data included age, sex, geographic region of Sweden, educational level, political party, political assignment for elder care, years as a responsible politician for elder care and work environment training received during the last two years (Table 1).

The investigative questionnaire was also developed in REDCap. The objective of the first part of the questionnaire was to understand the responsible municipality politicians’ self-rated knowledge regarding the work of the different key elder care professions in both special housing and home care contexts. The politicians were asked “*How do you grade your knowledge of the work of the following professions?*” [(1) *Care assistant within special housing*; *Care assistant within home care*; (2) *Assistant nurse within special housing*; *Assistant nurse within home care*; (3) *Registered nurse within special housing*; *Registered nurse within home care*; (4) *Physiotherapist within special housing*; *Physiotherapist within home care*; (5) *Occupational therapist within special housing*; *Occupational therapist within home care* and (6) *First-line manager within special housing; First-line manager within home care*] and asked to grade their level of knowledge as *Very high*, *Fairly high*, *Rather low*, *Very low* or *Do not know*.

The objective of the second part of the questionnaire was to measure the municipality politicians’ self-rated knowledge regarding their accountability for the work environments of the different key professions in elder care. The politicians were asked to grade their levels of knowledge as *Very high*, *Fairly high*, *Rather low*, *Very low* and *Not at all* in regard to the following statements: (1) *I have accountability for the work environment of the elder care staff*; (2) *According to the Work Environment Act, I have accountability as an employer for the staff’s work environment in elder care*; (3) *I know what can cause mental health problems among staff in elder care* and (4) *I know what can promote a healthy work environment in elder care*.

After the development of the draft questionnaires, the first and second authors research centre CTA (Centre for Work Life Studies) and the representative unions for the various key professions provided feedback on the content and distribution plans. Five politicians provided feedback after testing the questionnaires. The feedback received did not necessitate changes either to the questionnaires or the distribution plans.

### Statistical methods

It was not the intention to use inferential statistical methods to assess municipality politicians’ self-estimated levels of knowledge but rather to use comprehensive descriptive statistics to describe and analyse their knowledge conditions. Therefore, collected questionnaire data were exported from REDCap into SPSS Statistics Version 29 and compiled into tables and bar charts for further descriptive interpretation and analyses. The tables and bar charts provide information about the municipality politicians’ self-rated levels of knowledge of the work of the key professions and their accountability for the work environments of the key professions within special housing and home care.

The tables regarding knowledge of the work of the key professions (Tables 2–4) and knowledge of the accountability for the work environments (Table 5) show results for each questionnaire question and are specified for the groups All (*N* = 81; board chairs, board vice-chairs, deputy board vice-chairs, board members and deputy board members), Chair (*n* = 45; board chairs, board vice-chairs and deputy board vice-chairs) and Member (*n* = 35; board members and deputy board members). The group All has one more participant than the sum of Chair and Member groups because one participant did not specify their board role. Each table shows the number and percentage of participants for each answer as well as for missing answers (missing values).

**Table 2:** Municipality politicians’ self-rated knowledge levels of the work of the key professions within special housing

Participants	Very high	Fairly high	Rather low	Very low	Do not know	Missing
Care assistant						
All	9/11.11	46/56.79	17/20.99	8/9.88	0/0.00	1/1.23
Chair	6/13.33	30/66.67	8/17.78	0/0.00	0/0.00	1/2.22
Member	3/8.57	15/42.86	9/25.71	8/22.86	0/0.00	0/0.00
Assistant nurse						
All	16/19.75	38/46.91	18/22.22	7/8.64	1/1.23	1/1.23
Chair	11/24.44	27/60.00	6/13.33	0/0.00	0/0.00	1/2.22
Member	5/14.29	11/31.43	11/31.43	7/20.00	1/2.86	0/0.00
Registered nurse						
All	11/13.58	36/44.44	23/28.40	10/12.35	0/0.00	1/1.23
Chair	9/20.00	24/53.33	10/22.22	1/2.22	0/0.00	1/2.22
Member	2/5.71	11/31.43	13/37.14	9/25.71	0/0.00	0/0.00
Physiotherapist						
All	5/6.17	26/32.10	34/41.98	15/18.52	0/0.00	1/1.23
Chair	4/8.89	18/40.00	18/40.00	4/8.89	0/0.00	1/2.22
Member	1/2.86	8/22.86	16/45.71	10/28.57	0/0.00	0/0.00
Occupational therapist						
All	5/6.17	28/34.57	31/38.27	16/19.75	0/0.00	1/1.23
Chair	4/8.89	19/42.22	16/35.56	5/11.11	0/0.00	1/2.22
Member	1/2.86	9/25.71	15/42.86	10/28.57	0/0.00	0/0.00
First-line manager						
All	13/16.05	38/46.91	20/24.69	9/11.11	0/0.00	1/1.23
Chair	12/26.67	24/53.33	8/17.78	0/0.00	0/0.00	1/2.22
Member	1/2.86	14/40.00	12/34.29	8/22.86	0/0.00	0/0.00

**Note(s):** Numbers and percentages are given for the participant groups All (*N* = 81), Chair (*n* = 45), and Member (*n* = 35) in the format *n*/%. One participant (*n* = 1) did not specify board assignment role and was therefore not included in either of the Chair or Member groups

**Source(s):** Authors’ own work

**Table 3:** Municipality politicians’ self-rated knowledge levels of the work of the key professions within home care

Participants	Very high	Fairly high	Rather low	Very low	Do not know	Missing
Care assistant						
All	8/9.88	48/59.26	17/20.99	7/8.64	0/0.00	1/1.23
Chair	6/13.33	31/68.89	7/15.56	0/0.00	0/0.00	1/2.22
Member	2/5.71	17/48.57	9/25.71	7/20.00	0/0.00	0/0.00
Assistant nurse						
All	14/17.28	44/54.32	15/18.52	5/6.17	1/1.23	2/2.47
Chair	11/24.44	28/62.22	5/11.11	0/0.00	0/0.00	1/2.22
Member	3/8.57	16/45.71	10/28.57	5/14.29	1/2.86	0/0.00
Registered nurse						
All	11/13.58	32/39.51	28/34.57	9/11.11	0/0.00	1/1.23
Chair	9/20.00	22/48.89	12/26.67	1/2.22	0/0.00	1/2.22
Member	2/5.71	10/28.57	15/42.86	8/22.86	0/0.00	0/0.00
Physiotherapist						
All	5/6.17	23/28.40	38/46.91	14/17.28	0/0.00	1/1.23
Chair	4/8.89	15/33.33	21/46.67	4/8.89	0/0.00	1/2.22
Member	1/2.86	8/22.86	17/48.57	9/25.71	0/0.00	0/0.00
Occupational therapist						
All	5/6.17	26/32.10	33/40.74	15/18.52	0/0.00	2/2.47
Chair	4/8.89	16/35.56	19/42.22	5/11.11	0/0.00	1/2.22
Member	1/2.86	10/28.57	14/40.00	9/25.71	0/0.00	1/2.86
First-line manager						
All	12/14.81	36/44.44	23/28.40	8/9.88	0/0.00	2/2.47
Chair	10/22.22	24/53.33	9/20.00	0/0.00	0/0.00	2/4.44
Member	2/5.71	12/34.29	14/40.00	7/20.00	0/0.00	0/0.00

**Note(s):** Numbers and percentages are given for the participant groups All (*N* = 81), Chair (*n* = 45), and Member (*n* = 35) in the format *n*/%. One participant (*n* = 1) did not specify board assignment role and was therefore not included in either of the Chair or Member groups

**Source(s):** Authors’ own work

**Table 4:** Municipality politicians’ self-rated knowledge levels of the work of the key professions within special housing and home care

Participants	Special housing	Home care
Care assistant		
All	55/67.90	56/69.14
Chair	36/80.00	37/82.22
Member	18/51.43	19/54.28
Assistant nurse		
All	54/66.66	58/71.60
Chair	38/84.44	39/86.66
Member	16/45.72	19/54.28
Registered nurse		
All	47/58.02	43/53.09
Chair	33/73.33	31/68.89
Member	13/37.14	12/34.28
Physiotherapist		
All	31/38.27	28/34.57
Chair	22/48.89	19/42.22
Member	9/25.72	9/25.72
Occupational therapist		
All	33/40.74	31/38.27
Chair	23/51.11	20/44.45
Member	10/28.57	11/31.43
First-line manager		
All	51/62.96	48/59.25
Chair	36/80.00	34/75.55
Member	15/42.86	14/40.00

**Note(s):** Numbers and percentages were calculated as the sum of the answer options Very high and Fairly high. Numbers and percentages are given for the groups All, Chair, and Member in the format *n*/%

**Source(s):** Authors’ own work

**Table 5:** Municipality politicians’ self-rated knowledge levels of accountability for the work environments of the key professions within elder care

Participants	Very high	Fairly high	Rather low	Very low	Not at all	Missing
Responsible for work environment						
All	36/44.44	22/27.16	7/8.64	2/2.47	13/16.05	1/1.23
Chair	23/51.11	14/31.11	5/11.11	1/2.22	1/2.22	1/2.22
Member	13/37.14	8/22.86	1/2.86	1/2.86	12/34.29	0/0.00
Legally responsible for work environment						
All	46/56.79	17/20.99	3/3.70	1/1.23	13/16.05	1/1.23
Chair	27/60.00	13/28.89	2/4.44	0/0.00	2/4.44	1/2.22
Member	18/51.43	4/11.43	1/2.86	1/2.86	11/31.43	0/0.00
Knowledge of causes of mental illness						
All	13/16.05	41/50.62	19/23.46	2/2.47	4/4.94	2/2.47
Chair	8/17.78	29/64.44	5/11.11	1/2.22	0/0.00	2/4.44
Member	5/14.29	11/31.43	14/40.00	1/2.86	4/11.43	0/0.00
Knowledge of how to promote healthy workplaces						
All	10/12.35	37/45.68	23/28.40	5/6.17	4/4.94	2/2.47
Chair	7/15.56	25/55.56	11/24.44	1/2.22	0/0.00	1/2.22
Member	3/8.57	12/34.29	12/34.29	3/8.57	4/11.43	1/2.86

**Note(s):** Numbers and percentages are given for the participant groups All (*N* = 81), Chair (*n* = 45), and Member (*n* = 35) in the format *n*/%. One participant (*n* = 1) did not specify board assignment role and was therefore not included in either of the Chair or Member groups

**Source(s):** Authors’ own work

**Figure 2:**
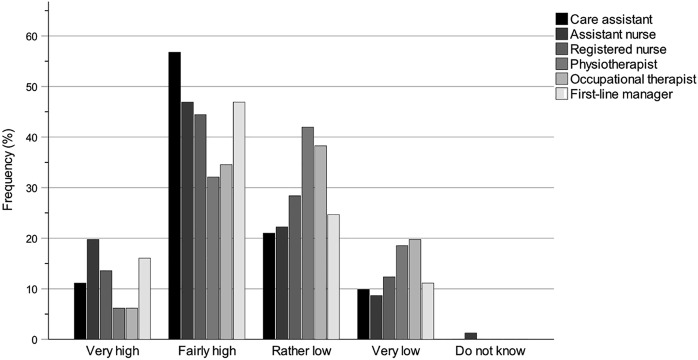
Municipality politicians’ self-rated knowledge levels of the work of the key professions within special housing. Percentages are given for the participant group All (*N* = 81). Source: Authors’ own work

**Figure 3:**
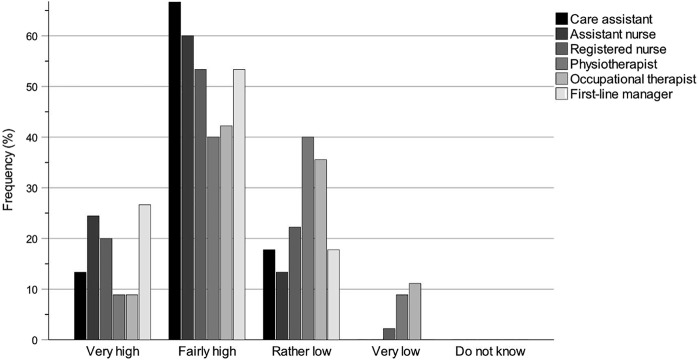
Municipality chair politicians’ self-rated knowledge levels of the work of the key professions within special housing. Percentages are given for the participant group Chair (*n* = 45). Source: Authors’ own work

**Figure 4:**
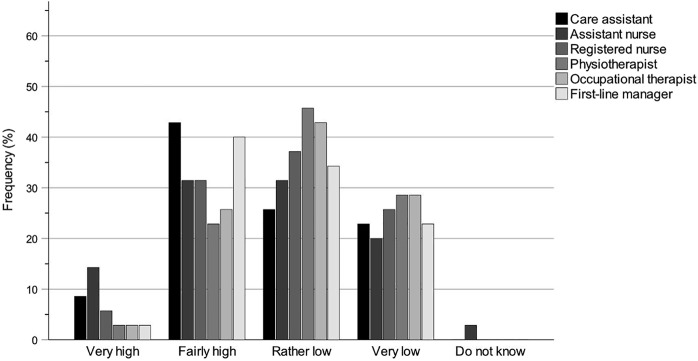
Municipality member politicians’ self-rated knowledge levels of the work of the key professions within special housing. Percentages are given for the participant group Member (*n* = 35). Source: Authors’ own work

**Figure 5:**
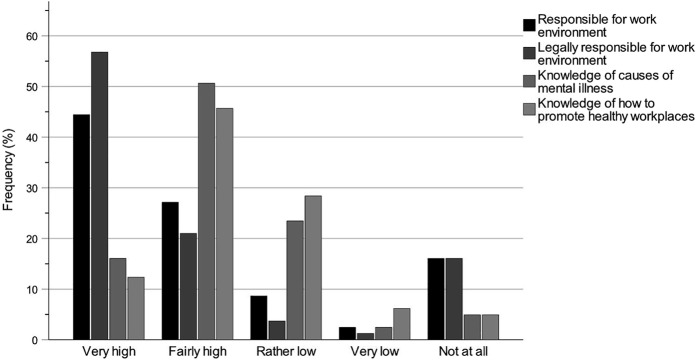
Municipality politicians’ self-rated knowledge levels of accountability for the work environments of the key professions within elder care. Percentages are given for the participant group All (*N* = 81). Source: Authors’ own work

**Figure 6:**
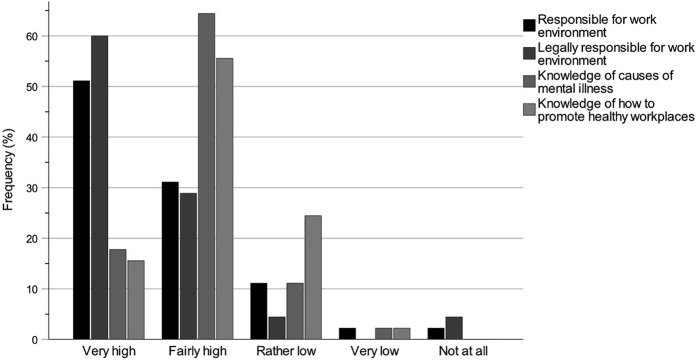
Municipality chair politicians’ self-rated knowledge levels of accountability for the work environments of the key professions within elder care. Percentages are given for the participant group Chair (*n* = 45). Source: Authors’ own work

**Figure 7:**
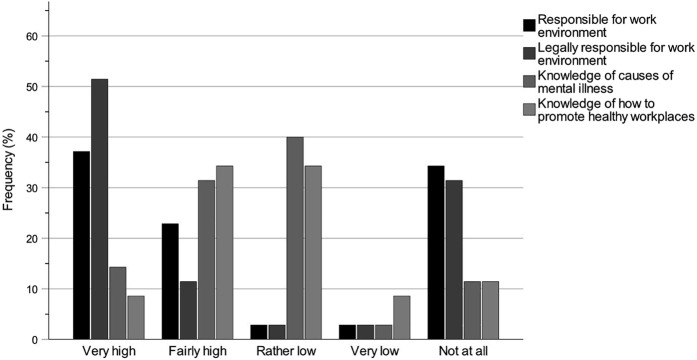
Municipality member politicians’ self-rated knowledge levels of accountability for the work environments of the key professions within elder care. Percentages are given for the participant group Member (*n* = 35). Source: Authors’ own work

The bar charts regarding knowledge of the work of the key professions (Figure 2 (All), Figure 3 (Chair) and Figure 4 (Member)) and knowledge of the accountability for the work environments (Figure 5 (All), Figure 6 (Chair) and Figure 7 (Member)) show percentages to visually complement the tables by illustrating the distributions of participants across answer options. Bar charts were only provided for special housing since the results for special housing and home care were similar.

## Results

A total of 81 municipality politicians agreed to participate in the study (Table 1). Political assignments for elder care were distributed as 23 board chairs, 12 board vice-chairs, ten deputy board vice-chairs, 23 board members, 12 deputy board members and one participant who did not specify board assignment. There were 51 women and 30 men with a mean age of 60.3 years (range 33–91 years). Participants were geographically distributed across Sweden’s three regions: the southern region of Götaland (*n* = 37), the central region of Svealand (*n* = 35) and the northern region of Norrland (*n* = 8). Approximately 73% of the participants (*n* = 59) had a university level education and about 27% (*n* = 22) had an upper-secondary education. Twenty-six participants belonged to the largest right-wing political party (The Moderates) and 20 belonged to the largest left-wing political party (The Social Democrats). The remaining 35 participants were distributed between other political parties (The Christian Democrats (*n* = 9), The Left Party (*n* = 11), The Centre Party (*n* = 4), The Liberals (*n* = 3), The Swedish Democrats (*n* = 3), The Green Party (*n* = 2) and local independent parties (*n* = 3)). Occupational status was represented by about 40 different professions, such as accountant, administrator, registered nurse, engineer, full-time politician, manager, sociologist, teacher, welder and retired participants (*n* = 19). The mean years as a responsible politician for elder care was 4.7 (ranging 1–30 years), and during the last two years, 18 participants received work environment training. See Table 1 for additional baseline demographic characteristics.

### Knowledge of the work of the key professions

Tables 2–4 show the municipality politicians’ self-rated levels of knowledge concerning the work of the different key professions within special housing and home care. Participants were divided into the groups of All (*N* = 81), Chair (*n* = 45) and Member (*n* = 35) to evaluate for differences by assigned role. The knowledge levels of each group about each key profession were estimated with numbers and percentages on a scale from Very high to Do not know. As can be seen, knowledge levels in special housing and in home care were similar (Table 4).

### Knowledge of the key professions within special housing

The distribution pattern of self-rated knowledge levels for special housing (Tables 2 and 4) among all participants was highest (Very high plus Fairly high) for care assistant (67.90%), assistant nurse (66.66%) and first-line manager (62.96%), lower for registered nurse (58.02%), and even lower for occupational therapist (40.74%) and physiotherapist (38.27%). There was a strong influence of the group Chair in this pattern where knowledge levels were high for assistant nurse (84.44%), care assistant (80.00%) and first-line manager (80.00%), lower for registered nurse (73.33%) and lowest for occupational therapist (51.11%) and physiotherapist (48.89%). Although the overall pattern was the same for the group Member, knowledge levels were lower across all studied professions: care assistant (51.43%), assistant nurse (45.72%), first-line manager (42.86%), registered nurse (37.14%), occupational therapist (28.57%) and physiotherapist (25.72%).

### Knowledge of the key professions within home care

Within home care, the distribution pattern of knowledge levels (Tables 3 and 4) among all participants was clearly similar to that of special housing. The knowledge levels of the group All were highest (Very high plus Fairly high) for assistant nurse (71.60%) and care assistant (69.14%), lower for first-line manager (59.25%) and registered nurse (53.09%) and lowest for occupational therapist (38.27%) and physiotherapist (34.57%). Similar to special housing, there was a strong influence of the group Chair in this pattern: knowledge levels were high for assistant nurse (86.66%) and care assistant (82.22%), lower for first-line manager (75.55%) and registered nurse (68.89%) and lowest for occupational therapist (44.45%) and physiotherapist (42.22%). Just as for special housing, the overall pattern was the same for the group Member with lower knowledge levels across all professions: care assistant (54.28%), assistant nurse (54.28%), first-line manager (40.00%), registered nurse (34.28%), occupational therapist (31.43%) and physiotherapist (25.72%).

### Knowledge patterns of the key professions within special housing

Since the results for special housing and home care are similar, bar charts were only compiled for special housing. Figures 2–4 illustrate the differences between the groups All, Chair and Member, and the knowledge patterns become even clearer by looking at the charts for the different key professions. The groups All and Chair have distributions skewed towards higher knowledge levels for care assistant, assistant nurse, registered nurse and first-line manager, whilst the distributions for physiotherapist and occupational therapist are nearly symmetrical or even skewed towards lower knowledge levels. The group Member has distributions that are nearly symmetrical for care assistant and assistant nurse, less symmetrical for registered nurse and first-line manager or even slightly skewed towards lower knowledge levels, whilst the distributions for physiotherapist and occupational therapist are also skewed towards lower knowledge levels.

### Knowledge of accountability for the work environments

Table 5 shows the municipality politicians’ self-rated knowledge levels of their accountability for the work environments of the different key professions. The knowledge levels of each participant group were estimated with numbers and percentages for each question on a scale from Very high to Not at all, and missing answers were included. The tables were complemented by clarifying bar charts, where the bar chart in Figure 5 shows the distributions for the participant group All across the answer options. Figure 6 shows the distributions for Chair, and Figure 7 for Member. The knowledge levels of the group All (Table 5 and Figure 5) were high for Q1: Responsible for work environment (Very high and Fairly high: 71.60%), Q2: Legally responsible for work environment (77.78%) and Q3: Knowledge of causes of mental illness (66.67%), but lower for Q4: Knowledge of how to promote healthy workplaces (58.03%). This pattern was strongly influenced by the group Chair (Table 5 and Figure 6; Q1: 82.22%, Q2: 88.89%, Q3: 82.22% and Q4: 71.12%), but different from the pattern of the group Member (Table 5 and Figure 7; Q1: 60.00%, Q2: 62.86%, Q3: 45.72% and Q4: 42.86%). The distributions for Member were not clearly skewed towards higher knowledge levels but were more evenly distributed between higher and lower levels: For Q1 and Q2, there were clear dichotomies in the distributions between higher and lower levels. For Q3 and Q4, the distributions were symmetrical but with tendencies towards lower levels of knowledge.

## Discussion

This study investigated the self-rated knowledge of municipality politicians responsible for elder care in Sweden and focused on two key aspects of their role: their knowledge of the work performed by key professionals who deliver elder care services and knowledge of their accountability as employers for the work environments.

### Lack of knowledge of professions in elder care

The findings reveal varying levels of self-rated knowledge among different political roles, and these are particularly distinct between board chairs (including board vice chairs and deputy board vice chairs) and board members (including deputy board members). Board chairs reported higher levels of self-rated knowledge across all questions concerning the key professions in both special housing and home care contexts and higher self-rated knowledge of work environment accountabilities.

The lowest levels of self-rated knowledge pertained to the professions of physiotherapists and occupational therapists regardless of the political board role. This aligns with previous qualitative research indicating a low degree of knowledge among municipality politicians in Sweden about these rehabilitation professions (Porter, 2022). The specific reasons for this lower knowledge are not well-documented, and the extent of the knowledge gap is not fully understood. Consequently, the lack of awareness among politicians about the significance of these licensed professions may lead to an underestimation of their crucial role in elder care, resulting in insufficient hiring to adequately address the demands in both special housing and home care settings. Indeed, research indicates that both physiotherapists (Carmona-Barrientos *et al.*, 2020) and occupational therapists experience significant work-related stress (Lexén *et al.*, 2020), and that this stress is to some extent due to insufficient rehabilitation staff in relation to the workload. This work-related stress is a contributor to occupational therapists’ considerations to leave their profession (Porter and Lexén, 2021). It is important to note that adequate staffing of occupational therapists and physiotherapists is associated with higher quality care for the elderly, including fall prevention (Livingstone *et al.*, 2019).

### The knowledge imbalance between political roles

The overall higher knowledge level among board chairs may be explained by their senior role on the board. However, according to the Swedish Local Government Act (SFS, 2017:725), all board members share collective and equal responsibility for oversight of their functional areas. The knowledge imbalance between board chairs and members should therefore not be accepted simply due to seniority. Nevertheless, it is crucial to recognise the fact that within the board organisation, 96% of the board members work part-time as politicians (Statistics Sweden, 2024) and as such have limited time to fulfil their political obligations. This time constraint may hinder their ability to stay updated across all areas of responsibility and to invest in a deeper knowledge of elder care activities.

The political role in Swedish municipalities includes being an employer, setting goals, directing the organisation and ensuring a safe work environment (Swedish Association of Local Authorities and Regions, 2012, 2019a, b). Regardless of the given political role on the board, understanding the extent of the politicians’ knowledge is important since Sweden’s elected political organisation(s) are accountable for elder care, and there is a high rate of sick leave rate among the staff (Swedish Social Insurance Agency, 2020b). Municipality politicians on the respective boards represent the municipality as the employer and are legally responsible for the work environments of all employees (Swedish Work Environment Authority, 2019, 2024). A lack of knowledge of different professionals’ work can impede the politicians’ ability to set appropriate goals and actions to improve their work environments. To address these issues and enhance elder care services, political leadership should have clear knowledge of the situation, its root causes and improvement measures as they set the budget and organisational goals. This would facilitate informed debate and action beyond specific party-political lines or local political interests. Previous qualitative research shows that despite being aware of the high absence rates, politicians may still lack understanding of the reasons for employee sick leave (Porter and Muhonen, 2021).

Swedish elder care is a politically led organisation, and therefore, the main responsibility for the work environment is the responsibility of the politicians who should ensure compliance with the legal frameworks. The methodology of SWEM is a widely used approach to work actively towards a compliant and healthy work environment through a collaboration between managers, employees and employee representatives for health and safety (AFS, 2001:1 Swedish Work Environment Authority). This study did not investigate employers’ knowledge regarding the specifics of the SWEM-wheel methodology; however, relevant and general questions regarding the professionals’ work and work environments were addressed. Legislation requires that risks need to be identified and actioned, which the employer is obligated to do (Swedish Association of Local Authorities and Regions, 2022). Within elder care, the municipality politicians’ set goals and allocate resources to their organisation. For example, providing additional training and support for first-line managers, ensuring adequate staffing levels and promoting a supportive leadership style can help balance job demands and resources, leading to better outcomes for both staff and the elderly in their care (Rahnfeld and Muller, 2023). Whilst day-to-day responsibility for the staff in elder care is delegated to the first-line managers, the main responsibility still rests with the accountable politicians, and therefore, it is essential for them to understand their organisation, the different professionals working within it and their work environments (SKR, 2022).

To ensure the delivery of high-quality elder care in Sweden, civil servants collaborate with politicians and provide necessary advice and information for informed decision-making (Swedish Association of Local Authorities and Regions, 2012, 2019a, b). Whilst civil servant support ensures a level of quality and consistency, previous qualitative studies highlighted a knowledge imbalance between politicians and civil servants (Porter, 2022). Greater knowledge among politicians would enhance knowledge equity and thereby enable them to ask more insightful questions, constructively challenge civil servants and make better decisions regarding budgets, goals and priorities. Understanding and making use of the SWEM-wheel methodology in the investigation of the work environment, assessment of risks and analysis, taking measures to eliminate or reduce risks, and finally, follow-up is compulsory. To turn this into use in practice, the awareness of professional roles in elder care would facilitate more effective collaboration between politicians and civil servants to enhance mutual understanding and improvement of the work situation.

### Methodological considerations

This study is part of a larger empirical study with a longitudinal single-group pre-post experimental research design (Shadish *et al.*, 2002). It employed a pretest–posttest methodology to assess the impact of a digital educational programme focusing on the organisational and social work environments within elder care, targeting accountable municipality politicians in Sweden. The current study explored participant knowledge before undertaking the digital educational programme. Although the study was not intended to use inferential statistical methods to assess municipality politicians’ self-estimated levels of knowledge, descriptive statistical methods were employed to analyse their knowledge conditions. In total, 1,556 emails, including reminders, were sent to potential participants, targeting all board chairs responsible for elder care across Sweden’s 290 municipalities as well as extending the invitation to board vice-chairs, deputy board vice-chairs and board members. A previous qualitative study on the same target group highlighted recruitment challenges, with 456 emails sent resulting in 41 participants (Porter and Muhonen, 2021; Porter, 2022). This study included 81 participants, indicating that the sample group may not fully represent all municipality politicians accountable for elder care in Sweden. The high percentage of part-time politicians, 96% (Statistics Sweden, 2024), might have contributed to the low participation rate and warrants further investigation in a qualitative study exploring whether the work environment of municipality politicians could explain recruitment challenges. Nevertheless, the 81 participants encompassed diverse ages and genders, came from various parts of Sweden, and had different academic and political backgrounds, which is considered a major strength of this study.

The results of this study are based on a questionnaire designed to assess the self-rated knowledge of municipality politicians regarding the work of the key professions in elder care within special housing and home care contexts. The questionnaire also assessed the politicians’ self-rated knowledge of their accountability for the work environments. The study focuses on the knowledge gap regarding the organisation and work environments and the legal responsibility as employers that was identified in previous qualitative research involving 41 municipality politicians responsible for elder care (Porter and Muhonen, 2021; Porter, 2022). The questionnaire itself was based on this knowledge gap identified by previous research and was specifically targeting the municipality politicians accountable for the Swedish elder care.

It could be argued that consistently distinguishing knowledge levels ranging from Very high to Do not know/Not at all across all participants is ambitious. This concern was thoroughly discussed within the research team and the research centre (CTA). Additionally, this methodological issue was addressed with the key professional unions and five politicians (who did not participate in the study). These individuals were emailed a link to the questions and provided feedback to the project leader. No changes were deemed necessary, as all participants who tested the questionnaire found the questions and the five grading options reasonable. Nonetheless, because the distinction between, for example, Very high and Fairly high could be problematic, these two options were combined in the results.

### Conclusion

This empirical study provides insights into the self-rated knowledge of municipality politicians responsible for elder care in Sweden and focuses on two key aspects of their role. Examination of their knowledge of the work performed by key professionals who deliver elder care services indicates differences in knowledge levels based on political roles. Board chairs demonstrate a higher overall degree of knowledge than board members. Regardless of political role, the level of knowledge was higher for the work of care assistants and assistant nurses and lowest for physiotherapists and occupational therapists. This lack of awareness may lead to an underestimation of their crucial role in elder care, insufficient hiring to adequately address the demands and high work-related stress among those delivering the service. Similar differences were found for politicians’ self-rated levels of knowledge regarding their accountabilities for the work environments as employers. The effective application of a methodology such as the SWEM-wheel to comply with legal requirements depends on a high degree of knowledge through awareness and curiosity, a proper allocation of responsibilities, and time for follow-up. These results highlight the knowledge imbalance between board chairs and board members and are significant because all board chairs and members share equal responsibility for the decisions made. Given the limited time board members have to fulfil their political roles, it is essential to consider this constraint when addressing their knowledge needs.
